# The presentation of self in the age of ChatGPT

**DOI:** 10.3389/fsoc.2025.1614473

**Published:** 2025-08-21

**Authors:** Nils Klowait, Maria Erofeeva

**Affiliations:** ^1^Transregional Collaborative Research Centre 318, Faculty of Mechanical Engineering, Department of Technology and Diversity, Paderborn University, Paderborn, Germany; ^2^Laboratory of Anthropology of Contemporary Worlds (LAMC), Faculty of Philosophy and Social Sciences, Institute of Sociology, Université Libre de Bruxelles (ULB), Brussels, Belgium

**Keywords:** artificial intelligence, ChatGPT, workplace automation, dramaturgy, impression management, Goffman

## Abstract

Contemporary debates about artificial intelligence (AI) still treat automation as a straightforward substitution of human labor by machines. Drawing on Goffman’s dramaturgical sociology, this paper reframes AI in the workplace as *supplementary* rather than *substitutive* automation. We argue that the central—but routinely overlooked—terrain of struggle is symbolic-interactional: workers continuously stage, conceal, and re-negotiate what counts as “real” work and professional competence. Large language models (LLMs) such as ChatGPT exemplify this dynamic. They quietly take over the invisible, routinised tasks that underpin cognitive occupations (editing, summarizing, first-draft production) while leaving humans to enact the highly visible or relational facets that sustain occupational prestige. Drawing on diverse sources to illustrate our theoretical argument, we show how individual workers, dramaturgical teams, and entire professional fields manage impressions of expertise in order to counter status threats, renegotiate fees, or obscure the extent of AI assistance. The paper itself, having been intentionally written with the ‘aid’ of all presently available frontier AI models, serves as a meta-reflexive performance of professional self-staging. The dramaturgical framework clarifies why utopian tales of friction-free augmentation and dystopian narratives of total displacement both misread how automation is actually unfolding. By foregrounding visibility, obfuscation, and impression management, the article presents a differentiated case for AI’s impact on the performative structure of work, outlines diagnostic tools for assessing real-world AI exposure beyond hype-driven headlines, and argues for a more human-centered basis for evaluating policy responses to the ‘fourth industrial revolution.’ In short, AI enters the labor process not as an autonomous actor, but as a prop within an ongoing social performance—one whose scripts, stages, and audiences remain irreducibly human.

## 1 Introduction

The following paper’s^1^ argument can be summarized as follows: we do not take the symbolic-interactional work of the professional world seriously enough – i.e. the way workers work to present themselves, their profession, and their professional output to others. We thus fail to appropriately assess the extent to which artificial intelligence (AI) has already stealthily entered many workplaces where cognitive labor is accomplished. By drawing on Goffman's (1959) dramaturgical perspective, we will highlight how partial – or what we will call ‘supplementary’ – automation is not just already ubiquitous, but an object of complex symbolic interaction performed by workers, professional groups, and perhaps even ‘humanity’ as a whole. By the end of the paper, we hope that the reader will be better equipped to understand a number of AI-related phenomena (such as the framing of Generative AI output as ‘soulless slop’, or people’s reluctance to talk about the role of AI in their work), have a more nuanced toolset for evaluating the current extent of workplace AI exposure, and have a more human-centric context for understanding both gloomy and utopian predictions about the future of work. While the paper aims to initiate a dramaturgical turn in discussions of workplace automation—and thus also has a sociological audience in mind—the paper’s analysis is relevant for a broader audience: every discussion of workplace automation that sidesteps ubiquitous and unavoidable dramaturgical self-presentation practices will be ill-equipped to formulate holistic evaluations, suggestions, and projections of AI’s impact on the social world. We thus hope to make the case for the importance of workplace dramaturgy for policy research, economic impact assessments, and works that aim to predict long-term impacts of AI on human labor.

The paper will start with a brief introduction to current (and projected) AI capabilities with respect to cognitive labor, followed by an introduction to Goffman’s dramaturgical framework, culminating with an attempt to show that what Goffman calls ‘impression management’ is a foundational element for the daily organization of work, which underscores how new technologies do not just automatically enter the human workplace, but rather are shaped by continuous human practices.

This paper is intended as a first-mover exploratory piece that primarily argues for the viability of a theoretical framing (dramaturgy) for understanding workplace AI exposure. Following Goffman’s footsteps, we will draw on a mix of field-inspired anecdotes, auto-ethnographic reflections, public vignettes, formal industry reports, and informal conversations with professionals who both deploy—and publicly defend—their use of artificial intelligence. At the same time, this paper itself was intentionally written with heavy AI use and serves as an auto-ethnographic example of AI justification—this sentence included.

## 2 An anecdote

Imagine being an early-career researcher at the department of sociology in 2015, at a small English-language university in Moscow. Nobody clamors to invite you to conferences yet, or is willing to join forces for lucrative grants. Between substantial teaching workloads, a publish-or-perish culture, and modest pay, you decide to make some money on the side. Lots of your Russian-speaking colleagues want their papers to be read internationally, and you speak both languages competently. So you reach out and offer editing and translation services—Russian to English. You are not a professional translator, you have never had any formal instruction on how to do it. But you speak the languages, know your specialist terms, and have an understanding of scholarly writing within your discipline.

Initial attempts are amateurish, but you iterate. Over the years, you improve your processes, gain a little bit of a reputation of being a good (and affordable) translator. Over time, you negotiate higher pay, better conditions—you can point at a portfolio of work already accomplished. You still struggle, you are not a pro; you work on weekends, in the evenings after regular work, and you struggle with untangling the crazy-long compound sentences common in Russian sociology. In 2016, Google Translate switches to a machine learning translation model. Barak Turovsky—then product lead—is a Russian speaker, and the Russian-English language pair is among the first to ‘go AI’. One evening you decide to play around with the system as you struggle with a particularly nasty set of Russian subclauses. Google Translate instantly and accurately accomplishes what you have struggled with for half an hour. You sit back, think about what this means for you, professionally. You feel numb—cumulative months of work are now trivial. You copy and paste an entire paper into the translator—the result is not bad, but you find mistakes that you would not have made. You breathe a sigh of relief and think about your next steps.

The above is an auto-ethnographic account of one of the co-authors and represents an archetypical case of AI exposure: not a high-level professional being wholesale replaced by some new ultra-smart non-human, but a tired gig worker suddenly discovering that a substantial part of their professional grind is automatable. They are still necessary for now—as editors, double-checkers, refiners, managers. But an AI has, without much disruption to their workflow, slotted into the gig worker’s labor process.

Gig work, incidentally, is on the rise—the modern worker should not expect to spend their working life slowly moving up the corporate ladder of a single company. Increasingly, they need to adapt to shifting market demands and technological changes, seek out retraining and deeply temporary opportunities, and generally live a life of precarity (Li, 2023). Artificial intelligence in ‘supplementary mode’ accelerates this trend: if a good chunk of a cognitive worker’s job tasks is suddenly automatable, they might be made redundant; some might argue that they can now do even more work, but not all tasks scale like this, and redundancy always remains a possibility. Others might argue—especially in marketing communiques—that AI will take the drudgery out of work, allowing the worker to focus on the truly human tasks (see Work Trend Index Annual Report, 2023). However—if we are honest with ourselves—a lot of human work may be called ‘drudgery’ while being an essential part of a professional identity. It seems unlikely that workers will be compensated for the rare and unpredictable moments of innovation, inspiration or brilliance once all the reliable everyday drudgery is automated away.

There are still narratives of ‘total replacement’ – robo-cops, robo-judges, grand talk of ‘AI will solve physics’ (Chuan et al., 2019; Marks, 2025; Stevens, 2024)—but redundancy is considerably more likely to come from consolidation rather than full displacement. Despite claims by Musk, Altman, and Co., even frontier AIs remain quite ‘dumb’ compared to the average human cognitive worker when evaluated holistically, rather than with narrow benchmarks (Loconte et al., 2023; Aghzal et al., 2025). Yet if we shift our attention to a contemporary AI’s capacity to chip away at the total workload of a given worker, we can appreciate that many persons can lose their job to systems that are substantially less performant than the average human.

## 3 The fourth industrial revolution

The phenomenon of automation has entered the spotlight of public discussion largely due to recent breakthroughs in AI. Since the introduction of advanced neural networks in the early 2010s, the fields of robotics, computer vision, and automation have made leaps that once seemed like science fiction. Today, AI can not only master a complex board game like Go but also describe images, summarize entire books, draw original artwork, and produce texts that rival human writing. With the rapid rise of large language models (LLMs) such as ChatGPT, these capabilities have accelerated further, enabling AI systems to assist with myriad human tasks—from composing emails to drafting legal briefs (Noy and Zhang, 2023; World Economic Forum, 2025).

Along with fascination at these new possibilities, experts have raised concerns about the implications for human labor, and large research groups have turned to the concrete economic disruptions that emerging automation may cause (Bick et al., 2024). Analysts note that technologies once deemed too context-dependent or creative for machines—like writing ad copy or summarizing detailed reports—are now regularly performed by LLMs in many workplaces (Mayer et al., 2025).

Recently the World Economic Forum (WEF) has already highlighted such concerns in its *Future of Jobs* report. Surveys suggest that, by 2030, AI and information processing technologies are expected to generate 11 million new jobs but also lead to the loss of 9 million, making them the currently most impactful technological trend (World Economic Forum, 2025; Thomson Reuters Institute, 2024). Although demographic and socioeconomic factors remain pivotal, the expansion of AI into everyday work practices is now recognized as a most divergent driver of job transformation. Another estimate from McKinsey suggests that up to 30% of total working hours both in Europe and in the US could be affected by AI-based automation in the coming years, with routine white-collar functions which involve repetitive and predictable tasks hit the hardest (Mayer et al., 2025). The estimates predict that workers in low- and middle-wage jobs are significantly more likely to face occupational transitions due to AI-driven automation: three to five times more likely in Europe and 10 to 14 times more likely in the US.

Many experts compare the scope of these changes to prior Industrial Revolutions, arguing that governments and businesses must act quickly to mitigate worsening inequality (Soroushian, 2024). If newly required skills are not cultivated, large segments of the workforce may be left behind. Using the metaphor of “the second machine age” (Brynjolfsson and McAfee, 2016) or the “cognitive industrial revolution” (Yee and Hoffman, 2024) helps frame LLMs and related AI as tools capable of automating “the unautomated”: namely, non-routine cognitive tasks. Earlier scholars often distinguished tasks as cognitive or manual, and routine or non-routine (Autor et al., 2003; Frey and Osborne, 2017). Historically, mechanized production first targeted manual, repetitive tasks; over time, computers automated routine cognitive tasks such as data processing. Now, generative AI expands that frontier to non-routine tasks, including original writing, coding, and summarization (Brynjolfsson et al., 2023; Noy and Zhang, 2023).

The following case study is a good illustration of the current AI capabilities that focuses specifically on creative, non-manual, and non-routine areas of human activity.

### 3.1 Automating the unautomated: the case of AlphaGo

Many things that previously seemed unautomated can now be done by a computer. Playing Go, for example. “The Western world has chess, but Go is an incomparably more subtle and intelligent game,” said Lee Sedol (Levinovitz, 2014), a 9-dan professional player with an Elo rating of around 3,500, a few years before his match with a Go-AI. Unlike chess, in Go the number of combinations of moves exceeds the current computing power of any machine. Indeed, the number of possible positions in Go exceeds sixteen billion, which eliminates the possibility of “mechanically” trying positions to choose the most efficient move. For this reason, the game was originally considered to be beyond automation for at least the next century.

In 2016, Google DeepMind’s system, AlphaGo, defeated Lee Sedol. Subsequently, in 2017, the algorithm defeated Ke Jie, the world’s top Go player at the time, with an Elo rating of 3,670. Using a combination of two neural networks trained on millions of master-level human games, AlphaGo managed to beat humans at a game that was, until then, the most difficult for a computer to master.

AlphaGo’s successor, AlphaGo Zero “becomes its own teacher. The system starts off with a neural network that knows nothing about the game of Go. It then plays games against itself … As it plays, the neural network is tuned and updated … and the process begins again … This technique is more powerful than previous versions of AlphaGo because it is no longer constrained by the limits of human knowledge. Instead, it is able to learn *tabula rasa* from the strongest player in the world: AlphaGo itself” (Silver et al., 2017, 354). After 3 days of “learning,” AlphaGo Zero surpassed the abilities of AlphaGo Lee, the AI that defeated Lee Sedol. After 21 days, it defeated AlphaGo Master, the AI that defeated Ke Jie. Finally, after 40 days, AlphaGo Zero surpassed all other versions of itself, reaching an Elo score of 5,185, while using only a fraction of the computing power of its predecessors (Silver et al., 2017). It is worth noting that AlphaGo Zero played at human beginner-level performances after only 3 hours of training, surpassing that level after 19 h, and reaching superhuman levels after approximately 70 h of training. Humankind has accumulated Go knowledge from millions of games played over thousands of years, collectively distilled into patterns, proverbs, and books. In the space of a few days, starting *tabula rasa*, AlphaGo Zero was able to ‘rediscover’ much of this Go knowledge, as well as novel strategies that provide new insights into the oldest of games.

The case of AlphaGo is a prime example of the automation of an ‘intellectual’ profession. It has shown that a computer is not only able to perform intellectual operations at the human level but can also surpass it. In parallel with these breakthroughs in board game automation, large language models such as ChatGPT have brought a similar transformation to creative and knowledge-based occupations (Achiam et al., 2024). These generative AI systems rely on architectures that learn from vast corpora of text, enabling them to produce human-like responses, draft complex written content, summarize large volumes of information, and even engage in tasks once considered exclusively in the human domain of expertise—such as writing essays, composing poetry, coding, or brainstorming new ideas (Bommasani et al., 2021). Much like AlphaGo’s tabula rasa learning reshaped our assumptions about what is “unautomatable,” LLMs raised questions about how language-related professions—such as writing, editing, translation, and research—may evolve when AI can mimic or surpass human capacities in these domains.

Similar to Go, human language was traditionally considered an area too contextually nuanced, culturally rich, and ever-changing for machines to grasp fully. As tools like ChatGPT continue to improve, they will likely provide increasingly accurate and contextually sensitive responses, contributing to the automation of a range of cognitive processes once deemed exclusively human. In turn, a growing body of research examines how these systems might transform the nature of intellectual and creative labor, from professional copywriting to advanced research support (Floridi and Chiriatti, 2020). Just as the AlphaGo victory prompted professional Go players to incorporate new strategies discovered by the AI, the widespread adoption of large language models has prompted knowledge workers—from journalists to legal analysts—to explore new workflows that integrate AI-generated drafts, summaries, and suggestions. The upshot is a shifting professional landscape where the boundaries between human expertise and algorithmic assistance blur. This trend involves not only an expansion of computational capabilities but also a reimagining of how intellectual work is carried out.

Yet certain frontiers seem to remain only partially automated—or entirely out of reach. Complex perception tasks in chaotic physical spaces still challenge robotics, though reorganizing warehouses has made automation more feasible. Creative intelligence also poses complications, as ‘genuine originality’ and cultural context rely on human insight and values (Noy and Zhang, 2023). Meanwhile, the high complexity of social intelligence tasks—mentoring, emotional support, or delicate negotiations—stalls fully autonomous solutions, and many people prefer interacting with humans in these contexts (World Economic Forum, 2025).

Consequently, the automation of intellectual tasks alone does not define this Fourth Industrial Revolution; rather, the key development is the rapid encroachment on non-routine tasks that once required distinctly human creativity or judgment.

In short, LLMs make it possible to automate high-level tasks with minimal explicit human instruction, drawing on learned patterns from colossal text datasets (Eloundou et al., 2023). While neither a guaranteed path to widespread displacement nor a simple recipe for productivity gains, these developments spur rethinking of how work is structured and how skills are valued.

In the next section, we will examine more closely how advances in AI alter the core logic of automation.

### 3.2 A new type of AI - on the way to automating mess

Human activity is disorderly. The same is not true for robots: to replace humans, they need to work with human ‘messiness’, rather than being penned in a clean and orderly space. This is a task that classic AI could not easily cope with. It was like an alien visitor to our world: it would need specialized equipment to survive in our atmosphere. Amazon’s automated warehouses are a good illustration. Amazon’s ‘Kiva’ robots are capable of navigating and sorting items in Amazon’s warehouse, making logistics considerably easy and more or less autonomous. Roomba-like robots navigate the warehouse and carry shelves of packages toward a designated area, where a human operator takes over. This system is made possible through a kind of ‘sterilization’: the warehouse itself is grid-based, aiding navigation. The floor is perfectly level and covered in QR-codes. The shelves that the robot lifts are specifically designed to allow the Kiva robot to safely go underneath. Most of the time, they move along a grid, essentially like a rook in chess: up, down, left, right. Every other object’s location is known to the robot navigating the warehouse. Things are radically different in the ‘human spaces’ of the warehouse, which are cordoned off by a yellow-black-striped marking on the floor: boxes are strewn about in no particular systemic arrangement, cardboard is unevenly stacked in differently-sized columns, there are random objects on the floor. It’s messy. Not a good environment for our alien visitor, hence the ‘barrier’ that separates these two spaces.

This type of automation can be called *substitutive* automation: it takes an automatable task and creates a hermetic environment where a robot may be capable of *replacing* a human. A relatively messy human warehouse is rationalized to the point of being habitable to a non-human. Disorderly aspects of the traditional warehouse are cordoned off and made inaccessible. Substitutive automation is also what is typically featured in science fiction (as well as discussions of automation in popular science magazines): a judge is replaced with a robo-judge, a cop with a robo-cop, a human worker with a robotic counterpart. The underlying logic is the idea that substitution is the dominant modality of automation, precisely because there will always be an area of ‘mess’, which either needs to be sterilized or isolated. Brynjolfsson and McAfee (2016) argued that in the “second machine age”—as cognitive tasks become automatable—technological progress shifts from complementing to substituting human labor: machines now drive, translate, analyse data, and even compose music. As they improve, they increasingly do the jobs rather than merely assist people.

Contrary to this view, we argue that technological development renders current era of automation more and more supplementary. This wave of automation is distinguishable by its ability to deal with human messiness. A good recent illustration is the difference between how people speak to a ‘smart speaker’ compared to ChatGPT. With smart speakers like Alexa, the users speak slowly, with clipped, emphasized enunciation, using generic phrases such as ‘Hey Alexa, play smooth jazz’ [for more work on ‘voice-shaped buttons’, see Klowait, 2017 and Albert and Hall, 2024]. An experienced Alexa-user will *know* that un-disciplined, ‘messy’ speech will result in miscomprehension by Alexa. Below are two transcripts of encounters with these different systems (as recorded by one of the co-authors):

**User**: Uhm… can you- uh… what’s that tune’s name… by Rick Astley maybe?

**Alexa**: I’m not quite sure how to help you with that.

In stark contrast, while *some* discipline is still required (notably, the voiced version of ChatGPT still struggles with turn-taking), it handles messier, procedural human speech without much of an issue:

**User**: Hi, um, I was, uh, I do not know, I was thinking of, you know that tune, I think, but it’s like by a guy called Rick Mastley or something like this? You know what I mean, right?

**ChatGPT Voice**: Oh yeah, you mean Rick Astley! Are you talking about “Never Gonna Give You Up”?

If we return to the alien metaphor, this would be the stage where the alien, having adapted to earth’s atmosphere, is finally able to explore on its own. As such, while this advance does not necessarily have to mean that Amazon Warehouses will now be more ‘humanly chaotic’, the line between human and nonhuman spaces has been blurred. At the minimum, we are now living in an age where robots can try and make sense of the mess. If they can make sense of human mess, there is no longer a good reason to confine robots to their sterilized realms. The containment has been breached.

This breach paves the way for *supplementary* automation. This does not merely mean that robots will be able to take more jobs – quite the opposite: robots will be increasingly able to enter in a quasi-symmetrical ‘partnership’ with humans, precisely because they are now able to be selective about the tasks that they can take over. While, in the substitutive view, automation involved a black box of human skills, robots of the new era can see a judge as a collection of perfectly automatable sub-components. And while it did not make sense to create the perfectly rationalized grid-based courtroom, there is nothing principally preventing the development of a robot that can sort through messily-arranged stacks of papers on a desk, or to sift through badly-formatted email conversations. Indeed, this view does not directly contradict Brynjolfsson and McAfee’s (2016) work. The authors stressed that, in certain scenarios, humans working alongside machines (e.g., AI-assisted doctors) could outperform either humans or machines alone. This is what they called “racing with the machine” (instead of “racing against”) — a hopeful model where people learn to use machines to enhance their productivity; however, this would require education, adaptation, and policy change. Our position complements rather than contradicts their account: the nature of the new type of AI itself creates an infrastructure to race with the machine.

Next, we turn to the question of how the multicomponent organization of the profession is reflected in discourses on AI automation.

### 3.3 Discourses on AI automation

The metaphor of the “industrial revolution,” often invoked to describe the latest stage of AI development, fosters a discourse of automation through the logic of substitution—machines taking the place of humans. From the era of the steam engine to the rise of information and communication technologies, each “revolution” has reconfigured employment by automating certain professions and ushering in new ones. Yet the public imagination consistently highlights the negative specter of job loss. Contemporary discussions of AI-driven automation adopt both dystopian and utopian tones. In the former, machines “steal” human jobs. From this standpoint, relatively expensive and error-prone humans can be replaced by computers that process large volumes of diverse data—medical records, legal documents, financial analyses—at lower cost. In the latter, a “post-labor society” is imagined: robots do the work while humans are free to cultivate themselves (see Neilson and Rossiter, 2019).

In both visions, humans are entirely supplanted by machines. Just as a door-closer took over a doorman’s role, AI today can ostensibly replace a wide swath of professionals. “Robot judges” and “AI cashiers,” once science fiction, have become plausible. But how accurately does this image reflect reality? Close examination of automation discourse reveals a focus on each profession’s visible tasks, at the expense of invisible routines. A “robot judge,” for instance, is portrayed primarily as handing down decisions, rather than managing all the backstage research, discussion, and contextual nuance integral to legal practice.

However, as current research on generative AI systems (e.g., ChatGPT, Gemini, DeepSeek, and others) suggests, real-world automation fits the supplementary mode more than its substitutive counterpart (Noy and Zhang, 2023; Ryazanov et al., 2024). An AI might take over specific tasks—like summarizing documents, coding routine functions, or drafting a first version of a legal brief—without supplanting the entire occupation. At the same time recent reports on the future of labor, such as WEF’s, portray the automation vs. augmentation, or human-machine collaboration, as a choice that is being made today on the level of development and/or implementation (World Economic Forum, 2025). This view, although it admits the complex multicomponent nature of human work, overshadows the fact that work is performed to make its more favorable components visible and to hide the unfavorable ones. The fact that three times more employees are using generative AI for at least one-third of their daily work tasks than their superiors assume (Mayer et al., 2025) may indicate that workers do not want to advertise the potential automation of a large chunk of their jobs.

## 4 Toward a workplace dramaturgy

Let us return to the Russian-English translator and ask ourselves this: after discovering the capabilities of the new Google Translate, will they reach out to former clients and inform them of the good news? Will they ask for less money in the future? Or will they, rather, do their darnedest to highlight Google Translate’s limitations, or avoid mentioning this topic altogether? Will they present themselves as a professional who knows what they are doing, or will they be open about their newly managerial role? In short, will they see these technological developments as a threat to their professionalism, and will they do anything about this? Will other translators? Will other humans in general? What will relevant professional associations do?

To examine how professional roles function from the perspective of employees themselves, we turn to Goffman’s (1959) dramaturgical framework, which explores how people make their work intelligible and valued for observers. Goffman uses the concept of “dramatic realization”—the process by which professionals vividly convey the qualities and attributes of their expertise. Communication, Goffman (1951) stresses, has its own demands, and the rights and obligations of a status are often poorly adjusted to them. As a result, when performing tasks, people have to do more than just do their jobs: they must also perform symbolic labor,^2^ i.e. demonstrate competence or authority as part of a social performance. Drawing on a dramaturgical/theatrical metaphor, Goffman analyses individuals as performers who enact roles to convey specific information to an audience in a given setting.

*“While in the presence of others, the individual typically infuses his activity with signs which dramatically highlight and portray confirmatory facts that might otherwise remain unapparent or obscure. For if the individual’s activity is to become significant to others, he must mobilize his activity so that it will express during the interaction what he wishes to convey.”* (Goffman, 1959, 30).

For instance, scientists often use specialized jargon as a way of dramatizing otherwise unseen intellectual effort. Surgeons, violinists, or boxers, by contrast, rely on extremely visible demonstrations of skill—performances that inherently display their mastery to an audience. In Goffman’s words (Goffman, 1959, 20), these professions are “wonderfully adapted” to public proof of excellence.

Some occupations, however, struggle with unfavorable social visibility. Nurses, for example, perform highly skilled observational and communicative work—coordinating with doctors, tracking patient symptoms—yet to the casual observer, their labor seems routine or low-skill. Because much of their expertise is invisible to patients, nurses lack straightforward ways to dramatize it. This mismatch between actual performance and public perception can diminish a profession’s social standing.

Thus, both invisibility and unfavorable social visibility necessitates additional effort on the part of a performer to demonstrate their own worth. In his book Goffman analyses the strategies developed by the Euro-American society to this end. One such strategy is the practice of “make-work”—the demand to appear constantly engaged with work tasks when observed (Goffman, 1959, 68). Offices with open layout have been designed with explicitly this idea in mind: in the presence of another’s gaze, employees must appear to be more focused on work tasks—as they will have to constantly “make work.” Luckily for workers, any space, no matter how open for observation it is, is divided into frontstage and backstage zones. As Goffman suggests, the *frontstage* is where individuals perform their roles in accordance with expected norms and decorum, carefully managing impressions for an audience. The *backstage*, in contrast, offers a space where the performance can be rehearsed or temporarily suspended (e.g., a kitchen for waitstaff). In workplace contexts, this division allows employees to navigate the pressures of constant visibility, even in environments that appear fully surveilled.^3^ In the same vein, the work process itself is usually hidden from customers in many spheres, so they would evidence only a polished result rather than the thorny path taken to get there.

Although Goffman’s dramaturgical framework had been criticized for an unfair portrayal of social actors as calculating hypocrites (Gouldner, 1970), Goffman (1959, 153–155) emphasizes that the dramaturgical perspective highlights one of the foundations of society. He identifies a “fundamental dialectic” (Goffman, 1959, 160) in social interaction: in order to act in a situation, people seek to “define” it, to understand what is going on, but they rarely have access to complete information. Instead, they have to rely on appearances—“cues, tests, hints, expressive gestures, status symbols, etc.” (Goffman, 1959, 160–161)—to interpret what’s going on. The less reality is directly accessible, the more individuals must focus on surface appearances. This makes social life *inherently performative*, as appearances become both the main source of understanding and a tool for impression management – the process by which individuals control or influence how others perceive them during social interactions.

It means that workers do not *only* stage their work efforts for the sake of maximizing profit. It means that they face two sets of demands: that of their professional duties and that of communication in the form of a dramatic realization. Without the latter, the former would not be possible, since defining the situation precedes any action. To be recognized as a responsible and hardworking employee, a candidate has to demonstrate their best qualities during a job interview—a necessity that explains the enhanced theatricality of such events—along with the hyperbolization of relevant virtues in CVs. Therefore, dramatization efforts cannot be discarded as superficial: they lie at the heart of human communication. The dramaturgical perspective transforms our view on automation prevalent both in public discourse and in many scientific reports: it is not only about what is actually done; it is also about what is made visible. The latter must account for the multicomponent nature of occupations that consist not only of routine and original tasks, but also of visible and invisible ones, with visibility having a positive or negative valence.

### 4.1 Dramaturgy, work and the changing politics of professional display

Goffman’s (1959) *The Presentation of Self in Everyday Life* launched what is now an expansive body of research into the theatrical aspects of social life. Organizational sociologists in particular have returned to his vocabulary to interrogate how credibility, status and authority are accomplished in the contemporary workplace. During the past two decades that literature has moved considerably beyond the coffee-houses and hotel lobbies of Goffman’s day, following actors into algorithmic labor platforms, performance-metric dashboards and ubiquitous video surveillance. Although the empirical settings have changed, the core insight remains: labor is always simultaneously practical and dramaturgical. Recent scholarship, however, has refined the mechanisms through which performances are accomplished.

Collinson (2003) in his synthesis of 20 years of his own organization research argues that late-modern surveillance-based organizations actively manufacture material and symbolic uncertainty—through short-term contracting, continuous appraisal and data-rich monitoring—and that workers, in response, adapt “survival practices” that bring about three distinctive subjectivities. *Conformist selves* embrace managerial scripts and ‘play by the rules’ in career building or distance themselves from it; *dramaturgical selves* perfect the art of concealing unfavorable information or exaggerating favorable given-off signs; and *resistant selves* express dissent through covert practices such as indifference, irony or sabotage. What matters for the present argument is that impression management is not optional ornamentation but structurally induced labor. The emotional and cognitive energy required to sustain a credible front – all expressive equipment (appearance, setting, manner) an individual uses to shape how they are perceived by others – becomes part of the job description, even if it goes unrecognized in formal task breakdowns (and therefore many AI impact assessments).

While Goffman assumed audiences that were physically co-present, contemporary employees increasingly stage themselves before algorithmic evaluators whose criteria are opaque and whose judgments are permanently archived. McFarland et al. (2023) capture this shift in a contextual framework that links “evaluative potential” to impression motivation and the nature of workplace interaction characterized by anonymity, permanence, synchronicity, and verifiability. On employment stage, for example, where information about a candidate is easily verifiable online, workers are less likely to adopt extreme or deceptive strategies such as directly lying on a job interview. In other words, the dramaturgical apparatus now includes server logs and spreadsheet cells as staging areas. This data-supported surveillance can also produce counter-intuitive elaborations of the old arts of concealment. When everybody can see that consultants are exhausted, for instance, the very display of exhaustion may be recast as proof of commitment. Bouwmeester et al. (2022) term this move “taint accentuation,” showing how elite management consultants highlight rather than downplay the undesirable aspects of their work, e.g., publicly rehearse stories of 48-hour work sprints and missed anniversaries, to reassure clients of their dedication while tacitly criticizing exploitative norms. Where earlier work on “dirty” occupations focused on neutralizing or hiding stigma, accentuation underlines that contemporary performers sometimes need to shine extra light on their hardship in order to satisfy evaluators who prize self-sacrifice. Indeed, this paper’s earlier narrative of overworked-ness might slot neatly into the latter practice.

While data-driven contexts have commanded substantial attention, a complementary set of studies has restored attention to embodiment and temporality. Rosengren (2019) revisits working-time practices and observes that employees who arrive early and depart late dramatize their dedication. Time, they argue, operates as a moral artifact whose public visibility substitutes for otherwise intangible measures of cognitive output. Bassetti (2021) shifts the analytical lens to multimodal interaction, showing how airport-security screeners rely on silent gestures such as “highlighting” suspicious areas on X-ray images. Expertise is conveyed through tacit coordination; speech resurfaces only when the expressive order threatens to collapse.

### 4.2 From individual to team performances

It might be easily overlooked that Goffman did not primarily speak about impression management as a personal matter, or a matter that is only relevant during an individual’s first encounter with people who do not know how to place that individual. Rather, this working consensus, and the accomplishment thereof, is most commonly a *team matter*. It is not (just) the individual waiter who sustains the demarcation between cultivated customer-facing mannerisms and the relaxed, oftentimes raucous, conduct behind the closed doors of the kitchen. This demarcation is sustained and threatened by all the waitstaff who is present to sustain or threaten the definition of the situation.

Moreover, this maintenance of demeanor and professionalism is not necessarily only a matter of situated (face-to-face) performance—threats to the consistency of a performance can come from elsewhere. As Goffman notes, a professional community will likely be keen to develop methods that foreground their members’ professional virtues and will downplay public scandals or talk of obsolescence. Goffman highlights the example of the pharmacist, who, in WWII, could be trained up within a much shorter timeframe than the years of schooling required during peace times. He argues that one reason for ‘years of schooling’ is not just functional necessity (time to acquire sufficient levels of competence), but rather part of a performance of professionalism. Similarly, it is unlikely that spending half a decade writing sociological essays (or reading them!) will be especially useful for the working practice of the field sociologist. Beyond abstract arguments about learning fundamentals and how to deal with complex texts, one more honest reason for the amount of schooling required for many intellectual forms of labor is the need to present a front of expertise not easily obtained beyond the walls of eminent specialist institutions.

For the purposes of this paper, the issue is not whether the ‘actual’ value of professional intellectual labor should be ‘cleaned off of the falsities of impression management’. Rather, we aim to stress that humans, as skilled symbolic operators, will present themselves, their work, and their professions in a strategic manner. They will highlight prestigious, or especially impressive, parts of their work, and will downplay those mundane aspects that are not as presentable (but which might crucially make up a considerable chunk of their daily labor). They will develop professional certifications, resist scandals, and will hide when a technology makes their work easier (and thereby possibly lowering the skill requirements to accomplish it). This will happen regardless of the nature of the work, because humans are capable of making judgments about the impact of their impression and thus adjusting what and how they present to serve their practical purposes.

There is no need to go far for a real-world example of such teamwork. One of the surprising results from the McKinsey Institute report (Mayer et al., 2025) is that surveyed employees, while using AI for their work tasks in amounts unimaginable to their employers, are also calling for greater support from leadership to help them adapt to the AI transition. Nearly half of the respondents expressed a desire for more formal training. They also highlighted the importance of incentives such as financial rewards and recognition in encouraging broader engagement with AI technologies. So, employees prefer not to disclose their extensive use of AI in the workplace to their employers, but, when asked directly, try to present such technologies as a tool which can additionally bring them financial and symbolic gains. Here we observe the workings of what Collinson (2003) calls dramaturgical selves.

Equally, we observe dramaturgical teamwork in the relationship between workers and leadership. The latter claim (World Economic Forum, 2025) that they plan to prioritize upskilling of their staff in response to AI exposure rather than reducing the existing workers with less relevant skills, but at the same time accelerating process and task automation and investing in technologies. 71% of employees (Mayer et al., 2025), at the same time, affirm that their organizations will develop and deploy these technologies ethically, despite recognizing that AI could significantly reshape or even replace aspects of their roles. They thus reveal their conformist selves (Collinson, 2003) showing dedication to the direction that their companies are willing to take. In the context of the survey, leadership presents itself as socially responsible while workers display trust in their management. Both teams try to emphasize their virtues directed to the general public, but the picture may vary significantly when such matters are discussed privately within the teams (cf. Orlikowski and Gash, 1994).

As noted previously, discourse on AI-based automation typically spotlights only the visible elements of a profession. A “robot doctor” is depicted diagnosing patients, “robot police” solving crimes, and “robot lawyers” drafting legal briefs, while the behind-the-scenes routines—organizing records, addressing nuanced interpersonal issues, or consulting with colleagues—go unmentioned. Popular media like films further reinforce these dramatized elements: Robocop is shown arresting suspects, not buried in paperwork. Because the invisible layers of professional work remain hidden from public view, an impression is created that AI can substitute the entire profession merely by replicating its most visible outputs.

In fact, modern automation does exactly the opposite—automating the invisible routines. The success of systems like ChatGPT is explained by the fact that they perform very well in the generation of standardized genres that define many contemporary jobs—emails, reports, summaries etc. Anthropologist Ilana Gershon (2023), following Graeber (2018), calls them “bullshit genres,” reflecting the symbolic value workers ascribe to the types of tasks that LLMs are ready to automate in certain professions. As studies indicate (World Economic Forum, 2025; Mayer et al., 2025), workers in low- and middle-wage service jobs are the most affected by AI exposure as their jobs include a large portion of such standardized genre production. Already having to cope with unfavorable social visibility, they now also have to mitigate the impact of potential automatization. It is not surprising that they prefer not to disclose their use of LLMs or frame them as imperfect assistants in the need of human supervision. Luckily to the affected, exactly because such routines are invisible, their automation is easier to hide. This may explain the results obtained by McKinsey Institute (Mayer et al., 2025) that three times more employees are using generative AI for their daily work tasks than their employers think.

LLMs become a new prop in this theater of work and alter the dramatic realization of professions. While analyzing the current extent of workplace AI exposure, we must look at a profession’s multicomponent structure that not only includes different types of tasks (manual and cognitive, routine and non-routine), but also visible, invisible and hidden elements that bear symbolic meaning to performing professionals. When the AI supplements invisible cognitive routines, as we have seen, there is a tendency among workers to obfuscate its day-to-day use. But if the AI takes over visible tasks or invisible tasks that are symbolically significant, human professionals might be pushed into backstage roles that lack immediate prestige or clarity. The result can be a profound shift in how an occupation is publicly perceived—particularly if its newly “visible” dimension no longer aligns with the core intellectual or relational skills that once defined it. In the last section, we examine the consequences of AI automation in various configurations of social visibility.

## 5 Large language models and the shifting dramaturgy of professional work

The foregoing sections portray contemporary organizations as interactional theaters in which insecurity, metricised surveillance and digital micro-stages continually reshape what must be displayed, to whom, and at what cost. Large language models such as ChatGPT enter the dramaturgical domain not as independent actors but as versatile props for human dramatic expression, i.e., a novel tool of impression management. Because they excel at processing and generating the very textual genres—e-mails, minutes, briefs, syllabi—that constitute the backstage routines of cognitive work, their arrival reconfigures the distribution of visibility and thus the logic of professional impression management. In this section we trace three interrelated consequences of LLM adoption: the hollowing-out of routine visibility, the multiplication of evaluative audiences and the redirection of performative effort toward affect and ethos.

LLMs demonstrate their greatest comparative advantage in tasks that are simultaneously mundane and cognitively demanding: summarizing voluminous documents, producing first-pass translations, drafting contract boiler-plate or generating synthetic literature reviews. The accomplishment of these tasks has long been invisible to lay audiences, yet it underpins the credibility of professions such as law, translation, journalism, or academic research. When an attorney presents a crisp precedent table in court, the dramatic realization of competence depends on hundreds of unseen hours of discovery; when a translator submits a polished text, clients infer mastery of both languages from the absence of surface errors rather than from direct observation of word-choice deliberations. By assuming this backstage labor, LLMs hollow out a crucial evidential substrate of performance. The immediate dramaturgical effect is to render traditional markers of diligence—stacked files, annotated drafts, late-night office lights—anachronistic. Such markers once signaled devotion and expertise in situations where substantive competence could not easily be assessed, echoing Rosengren’s (2019) observation that working-time display functions as a moral proxy for productivity. When a lawyer can now instruct an LLM to “summarize discovery set A versus B” in minutes, the visible correlate of that painstaking work evaporates. Professionals must therefore search for replacement cues capable of reproducing a semblance of industrious depth.

LLMs not only displace certain backstage tasks; they also act as audiences in their own right, because they evaluate, rewrite and sometimes fact-check the texts they generate. The performer thus addresses multiple layers of scrutiny: the model’s internal scoring function, the platform’s content filters, the human client or supervisor and, potentially, an external fact-checker who may employ yet another model. These layered audiences intensify material and symbolic insecurities Collinson (2003) described. A polished memo produced with AI help may sail through managerial approval only to be flagged weeks later by a plagiarism detector or a governance audit. McFarland et al.’s (2023) contextual influences on impression management become newly salient. The asynchronicity between performance and evaluation makes every document a potential time-bomb. Consequently, workers engage in preventive facework reminiscent of Goffman’s “make-work” practices, but updated for the age of digital traceability: they may archive prompt histories, cite model versions, or interleave human commentary that reiterates professional norms.

As LLMs threaten to commodify linguistic craft, the locus of distinctive human contribution may partially shift toward affective and ethical domains. This redirection parallels the trajectory charted by Bassetti (2021) in airport security, where tacit bodily coordination becomes the valued site of expertise once technology routinises detection. In professional services the analogous premium now attaches to judgemental empathy: the capacity to translate client anxieties into bespoke prompts, to sense when factual hallucinations might slip through, or to shoulder accountability for decisions that no model can own.

Such elevation of ethos has two dramaturgical consequences. First, it privileges overt declarations of responsibility—“I have personally verified all citations”—over demonstrations of solitary craft. Second, the turn to ethos invites accentuation of vulnerability rather than mastery. Consultants who once boasted of sleepless nights might now emphasize their role as conscientious stewards who prevent AI error from harming clients. Yet this realignment is unstable. To the extent that empathy and judgment appear intangible, they risk being dismissed as vague “fluff,” much as nursing’s relational labor has long suffered unfavorable visibility. The challenge facing professionals, therefore, is twofold: to cultivate genuinely human capacities that complement model outputs, and to dramatize those capacities in ways that observers recognize as valuable. Without tangible props—or with props that algorithms can easily mimic—this task may prove harder than sustaining the old symbolics of desk-side piles and annotated drafts.

The cumulative pattern sketched above substantiates the claim advanced in this paper: AI adoption in knowledge work unfolds primarily as *supplementary automation*. Rather than replacing workers, LLMs infiltrate the backstage, remove labor whose visibility served as indirect evidence of expertise, and thereby oblige humans to invent new forms of performance that re-establish the grounds of professional trust. This is not a friction-free transition. New insecurities arise around disclosure and authenticity; new forms of competency labor emerge in the management of digital traces; and new fault-lines of status open between workers able to dramatize empathy convincingly and those whose roles remain tethered to tasks now readily modeled.

Understanding these dynamics is essential for both empirical research and policy design. Analytically, it cautions against treating AI exposure as a simple task-substitution ratio; the dramaturgical functions of tasks must be considered alongside their technical content. Practically, it suggests that organizations which mandate AI use without providing scripts for its ethical and affective integration may inadvertently erode the very professionalism they hope to augment. The dramaturgical framework therefore offers both diagnosis and prescription: it reveals why the visible surface of work can remain stubbornly human even as its invisible machinery becomes algorithmic, and it highlights the kinds of support workers need—recognition, narrative resources, spaces for controlled disclosure—if they are to stage credible and sustainable performances in the age of large language models.

## 6 Conclusion

This article has been a thought experiment rather than an exhaustive empirical study or a heavyweight theoretical intervention. Our goal was to offer what Blumer (1954) once called “a sensitizing concept” for understanding the dramaturgical transformations rippling through contemporary workplaces in the wake of what we might term the *grass-roots use artificial intelligence*—AI that enters organizations not through multi-million-dollar integration projects, but silently, through browser windows, smartphone apps and newly installed plug-ins for the software we already use. Word processors now autocomplete sentences; slide decks suggest layouts; e-mail clients draft polite replies. The transformation is quotidian, iterative and, above all, easy to overlook precisely because it hides behind familiar interfaces.

The argument unfolded from two linked observations. First, the world of work is not exempt from symbolic practice. Professionals, managers and gig workers alike spend significant time performing what Goffman called *dramatic realizations*: the labor of making competence and dedication visible to relevant audiences. Second, these performances are often deliberately obfuscated. The backstage of work—late-night Excel clean-ups, frantic inbox triage, half-finished arguments in the margins of a PDF—remains out of sight even as it sustains the polished front stage that clients, students, or patients encounter. Grass-roots AI now infiltrates that backstage, automating or accelerating tasks that once supplied indirect evidence of expertise. The result, we have suggested, is a profound redistribution of visibility, responsibility, and anxiety.

We cannot analyze this phenomenon without inhabiting it. Large sections of the present article were drafted, reformulated or annotated with the ‘help’ of ChatGPT o3—the newest widely available model at the time of writing. We undertook the ‘collaboration’ in part as a methodological experiment and in part because, like so many academics, we are stretched thin by teaching loads, administrative service, and other obligations. The experience was revelatory and unsettling. We found ourselves hoping the model would hallucinate, so that our corrective labor could restore a sense of authorship. We checked citations, worried about unintentional plagiarism and fretted over whether the prose still “sounded like us.” We made sure that nothing was copy-pasted, and that AI contributions were only initial drafts that augmented existing writing – and we made sure to point this out in the text. More troubling was the creeping question of intellectual contribution. If an LLM can ingest 20 PDFs on dramaturgy and emit a coherent literature review in 30 s, what remains for the human scholar? Beyond existential fears of replacement, we discovered an intense dramaturgical pressure. Were we now simply managers supervising a non-human subordinate? If so, how could we dramatize our supervisory role—our “responsibility,” our “editorial judgment”—in a way that preserved professional identity? Indeed, might it not be easier to inflate the LLM’s capacity to hallucinate and dismiss it outright as a viable workplace tool?

As routine craft becomes commodified, the locus of distinctively human contribution might partially shift toward affective and ethical domains—listening to a distressed patient, framing a narrative, shouldering accountability. These are not new tasks, but they assume new prominence when the mechanical aspects of writing or calculation are outsourced. The physician emphasizes empathic bedside manner; the journalist foregrounds field interviews; there are people who call themselves ‘prompt engineers’ (or, to use an even more unsettling term – ‘AI whisperers’). Yet, as nursing has long demonstrated, relational labor often suffers unfavorable visibility. The risk is that what remains human becomes, ironically, what institutions undervalue.

A final anecdote: we once ran an AI workshop with mid-level managers and departmental heads; as sociologist and AI scholars, we explicitly adopted a critical and reflexive position on AI, and structured the workshop activities accordingly: The session invited participants to detect AI-generated text and to reflect on their own likely use of such tools under ordinary work pressures. During the exercise, everyone professed unwavering vigilance – for example, they would *always* be mindful of LLM hallucinations and check every source manually. When we suggested that fatigue, deadlines, and ambient chaos might erode that vigilance, they doubled down: *They* would, *for sure,* remain watchful, though perhaps *their subordinates* might lapse. So, even in a setting explicitly devoted to critical reflection, participants engaged in spirited dramaturgy. They strived to present themselves as responsible stewards of technology, morally distinct from the imagined “careless worker” who might succumb to convenience, mirroring techbro-led AI replacement discourse of ‘lazy workers’. The moment revealed how difficult (if possible) it is to reach a meta-level beyond performance. Talking about AI is itself a performance, entangled in status claims, managerial authority, and latent fears.

The current grass-roots AI use has a curious quirk that intersects with dramaturgy: Large language models are genre machines trained on front-stage data: published articles, polished press releases, publicly visible social-media posts. They therefore reproduce front-stage stylistics with uncanny fidelity while remaining largely blind to backstage coordination, emotional labor and informal troubleshooting. When senior executives hail GPT-style systems as replacements for engineers or researchers, they may be grossly underestimating the dramaturgical distortions of the training data that underpins LLMs. By definition, these models do not ingest the messy, private repositories in which most professional work actually occurs. Consequently, organizations that lay off workers on the assumption that “ChatGPT can do it” may discover too late that they have amputated the invisible muscles holding the skeleton together, as they may have unwittingly excised structurally important symbolic labor.

If it will soon be possible to transform a three-sentence prompt into a publishable article, scholarly and professional communities must devise new conventions for signaling labor and responsibility. Possibilities include journals requiring a “prompt provenance” section, universities teaching prompt literacy alongside citation ethics, or professional bodies demanding signed attestations of human oversight. These innovations would inevitably become props in a revised dramaturgy of expertise, allowing audiences to continue inferring diligence from visible traces even when backstage routines are machine-assisted. At the same time, we foresee a class-wide and perhaps species-wide dramaturgical project: dethroning hype-laden narratives of machine supremacy by reclaiming the value of ‘real’ human labor. Outrage at AI-generated art (‘this is disgusting, soul-less AI slop’), we argue, is not only about (the very valid and real) expressions of job precarity or genuine moral indignation. It is also a symbolic struggle to preserve the uniqueness of human creativity. Detecting ChatGPT-cliché wording like “delve into the tapestry” becomes a folk method of boundary-work, marking a text as machine-made and therefore deficient. Whether such markers will hold as models improve is uncertain, but the attempt itself is dramaturgically significant. It is furthermore notable that hallucinations and genre cliches are highlighted, while lazy writing and human errors are marked as exceptional or, more realistically, remain unmentioned.

Our exploratory argument raises empirical questions. How exactly do professionals of various backgrounds re-allocate time between substantive analysis and prop maintenance? What new genres of credibility emerge around AI disclaimers, and how are they policed? How does the redistribution of labor affect inequalities of gender, race and class, given that relational work has historically been feminized and undervalued? Ethnographic, longitudinal, and systematic empirical studies are urgently needed to trace these shifts. Indeed, much like existing studies of workplace dramaturgy highlight a variation in performances across sectors, one key upcoming line of inquiry would be the study of how *different* professional fields stage their dramaturgical selves in the context of artificial intelligence. While this paper was intended as more of a first-mover meta-reflexive experiment, we hope that subsequent empirical work will follow; this would enable cross-cultural and cross-professional comparisons and perhaps also highlight how dramaturgical performances systematically differ across intersectional lines.

Equally pressing is the normative question of regulation. If intellectual work increasingly depends on hybrid human-AI teams, labor law and professional licensure must grapple with issues of authorship, liability and remuneration. Without intervention, the danger is not a jobless future but a future of degraded work in which humans perform high-stakes supervision without commensurate recognition or pay.

Throughout this project we have tried—sometimes unsuccessfully—to step outside our own dramaturgy. Our public admission of AI ‘assistance’ is both a methodological disclosure and a bid for ethical capital. Recognizing this, we conclude on a deliberately modest note. Dramaturgy is not a superficial layer we can peel away to reveal “real” economic or technical processes. It is part of the infrastructure of cooperation, status and meaning-making that sustains professional life. Grass-roots AI does not abolish that infrastructure; it remodels it, often in ways still poorly understood. Sociological attention to the politics of visibility is therefore indispensable – it is a primary domain for the human-led contestation of AI. Only by tracing how people renegotiate what must be shown, what may be hidden and what can safely be entrusted to machines will we understand the full implications of artificial intelligence for labor, identity, and the fragile art of getting recognized as competent in an increasingly automated world.

## 7 Meta-conclusion

As was outlined in its beginning, and performed in the Acknowledgements section of this paper, we aimed to use all the state of the art AI systems available at the time of writing. This allowed us to not only write about—but also perform—our point about AI-induced changes in workplace dramaturgy of ‘intellectual’ professions. We would like to use this section to ‘break the fourth wall’ and reflect on these experiences. For this—and only this—section, we have disabled all AI systems, including spell-checkers, style advisors, feedback-givers, rewriters, etc.

During the creation of this paper, we asked LLMs to do literature reviews for us, rewrite sections of the paper, give feedback on existing writing, give summaries of relevant papers, suggest titles, check our spelling, translate sections that were initially written in another language, and transcribe our words as we dictated them, removing uhmms and ahhs.

Some of these things seemed dramaturgically ‘safer’ than others. The built-in style advice and spellchecker of Microsoft Word, for instance, felt quite professional – it highlighted text that we wrote and presented a menu of potential corrections from which we made a conscious selection. Even seasoned professional writers like us make spelling mistakes and allow some grammatical incongruencies – we work so hard, after all, and so much! It is almost a point of professionalism to have initialized this double-check on our work. Indeed, surely this paper will be spellchecked by others during the typesetting phase, so it’s not like we are doing on top of what is already comfortably part of the scholarly process. Similarly, finding additional literature—which we then read, naturally—could also be chalked up to professional due diligence – we are just checking our work and making sure that we did not miss anything. The ‘safest’ case here was the dictation transcription – we could go through the transcript and make sure that we ‘owned it’ fully, and that the AI only touched epiphenomenal components of our dictated words.

On the other side of the spectrum were the systems that did our work *for* us – writing entire sections and formulating arguments, analyzing and summarizing papers, spitting out (even now we cannot avoid articulating a certain distaste) full literature reviews. Here the procedure for organizing a professional ownership simply did not work, at least if we are honest – there is nothing collaborative about ‘Computer, write me a literature review on existing Goffmanian studies of workplace dramaturgy!’ and pressing the ‘submit’ button. For such cases, only concealment and transformative work seemed to be face-preserving pathways: we could take the ‘raw’ literature review and, sighing at the undergraduate-level perfunctory analysis the system performed, try to salvage and elevate the text to our professional standards. In short, we could take ownership of AI work by highlighting our own contributions to it.

Alternatively, if the system did not produce outright nonsense (which it, thankfully, usually still does), we simply could try to obscure the fact that it was AI-written by removing obvious ‘tells’ – such as the tendency of LLMs to over-use dashes. This mirrors Goffman’s (1959, 114) account of factory work dramaturgy:

“If a factory worker is to succeed in giving the appearance of working hard all day, then he must have a safe space to hide the jig that enables him to turn out a day’s work with less than a full day’s effort”.

This also highlights a temporal dimension. In the context of the paper, the performance of professionalism starts with the paper’s production (the work visible to faculty colleagues – being visibly busy, not being seen with ChatGPT open, cultivating an image of justifiable ChatGPT-use, etc.), to its publication (interaction with editors and peer reviewers—writing meta-conclusions, practices of distancing and muddling of concrete contributions of non-humans to a professional task, etc.) to its ultimate appearance to the readers (clean, with clear authorship, with the reputation of the journal, the authors, the institution, and the field paying some manner of trust forward). At each of these steps, the use of AI could be obfuscated, muddled, creatively transformed, un-creatively transformed or, in the case of spellcheckers and automatic suggestions, could go unnoticed. Each of these stages are rife with future scandals, university-wide policy initiatives, AI-user witch-hunts, the development of dramaturgically advantageous professional standards, and semi-performative acts of responsibility-taking (such as our Acknowledgements). In Goffman’s (1959, 44) words, and with the notable reframing of the previously mentioned concept of drudgery:

“[…] In those interactions where the individual presents a product to others, he will tend to show them only the end product, and they will be led into judging him on the basis of something that has been finished, polished, and packaged. In some cases, if very little effort was actually required to complete the object, this fact will be concealed. In other cases, it will be the long, tedious hours of lonely labor that will be hidden. For example, the urbane style affected in some scholarly books can be instructively compared with the feverish drudgery the author may have endured in order to complete the index on time, or with the squabbles he may have had with his publisher […].”

The question of power differentials emerges even here. While we are taking a modicum of risk in performing this experiment ‘in the open’, this openness cuts only one way: we are somewhat safe in disclosing our AI-use in this paper, but we certainly would not be in the position to highlight and problematize AI use further down the chain of the editorial process. This creates the situation where there is a hidden double standard of AI-use scrutiny: as authors, we will do our best to hide, obfuscate, meta-reflexively ‘own’ our AI use. If we find ourselves in the role of peer reviewers, we may see very little dramaturgical threat in copy-pasting the to-be-reviewed paper into a ChatGPT window and submitting that as a review after only light edits. This can change, but it matters that the front and back stages of AI use can be differently proportioned and thus, differently scrutinized. Given the fact that artificial intelligence systems are largely controlled by data-hungry corporations that operate with only little transparency, the extractive dimension is manifested even in such a low-stakes area as sociological theorizing. AI is not simply a thing that people may choose to use, but can also be a thing that happens *to* them externally. Challenging, resisting, or even merely mentioning this fact is dramaturgically—and professionally—costly. This circumstance creates this curious dynamic where individual scholars may need to engage in labor-intensive dramaturgical work to mitigate threats from AI use, while individual publishers—through their review systems, incentive structure, contractual agreements, and package deals with AI corporations, may deploy AI at a much grander scale, with only a modest dramaturgical cost. More generally, this means that the recognition of the ubiquity of the dramaturgical dimension in workplace automation does not mean that the face threats and requisite performative mitigation needs are evenly distributed. It appears highly probable that structural inequalities across gender, race, age, class will modulate dramaturgical cost associated with AI, and may represent another shadow cost of work.

As with our Google Translate anecdote in the beginning – LLMs are still incapable of replacing us as scholars. Indeed, we felt a certain sense of near-moral superiority when the top-performing LLMs would produce writing that would barely pass our expectations for second-year undergraduates. Yet, these systems did arguably make our job easier in a way that threatened authorship and professionalism; much like the worker’s helpful jig, there is a threat to being discovered as AI-reliant, and a pressure to anticipate embarrassing face threats. For sociologists of artificial intelligence, much of this embarrassment can be folded into meta-reflexivity (as we are doing here), and much of the threat can be relativized by pointing at an LLM’s laughable attempt at sounding scholarly (rather than reproducing the literary of a corporate presentation), its tendency to hallucinate, and through public denouncements of AI use.

Yet, what of the second-year sociology undergraduates these systems are threatening? How will *they* dramatize their use going forward, and what will we do when these systems finally graduate?

## Data Availability

The original contributions presented in the study are included in the article/supplementary material, further inquiries can be directed to the corresponding author.

## Footnotes

^1^This paper is a continuation of an older paper (Klowait and Erofeeva, 2019) that was written prior to the emergence of LLMs, but after the emergence of powerful convolutional neural networks. Much of the early argument is here extended and applied to a much broader domain of human cognitive labor.

^2^In what follows, we refer to “symbolic” to mean performative and communicative work by which professionals convey competence and credibility, in contrast to technical tasks they have to perform to do their jobs.

^3^The extreme example would be mental health hospitals or prisons. Goffman (1986) shows that even in “total institutions” asylums find ways to establish backstage zones, even if they are not permanent or entirely physical.
